# Changes in the total leukocyte and platelet counts in Papuan and non Papuan adults from northeast Papua infected with acute *Plasmodium vivax *or uncomplicated *Plasmodium falciparum *malaria

**DOI:** 10.1186/1475-2875-7-259

**Published:** 2008-12-18

**Authors:** Walter RJ Taylor, Hendra Widjaja, Hasan Basri, Colin Ohrt, Taufik, Emiliana Tjitra, Samuel Baso, David Fryauff, Stephen L Hoffman, Thomas L Richie

**Affiliations:** 1 The US Navy Medical Research Unit no. 2, Jakarta, Indonesia; 2 Dept. of Tropical Medicine, Tulane University School of Public Health, New Orleans, USA; 3 Division of Experimental Therapeutics, Walter Reed Army Institute of Research, Washington DC, USA; 4 Indonesian Naval Hospital, Jayapura, Papua; 5 Centre for Health Research and Development, National Institutes of Health, Jakarta, Indonesia; 6 Rumah Sakit Umum, Jayapura, Papua, Indonesia; 7 Naval Medical Research Center, Bethesda, Maryland, USA

## Abstract

**Background:**

There are limited data on the evolution of the leukocyte and platelet counts in malaria patients.

**Methods:**

In a clinical trial of chloroquine vs. chloroquine plus doxycycline vs. doxycycline alone against *Plasmodium vivax *(n = 64) or *Plasmodium falciparum *(n = 98) malaria, the total white cell (WCC) and platelet (PLT) counts were measured on Days 0, 3, 7 and 28 in 57 indigenous Papuans with life long malaria exposure and 105 non Papuan immigrants from other parts of Indonesia with limited malaria exposure.

**Results:**

The mean Day 0 WCC (n = 152) was 6.492 (range 2.1–13.4) × 10^9^/L and was significantly lower in the Papuans compared to the non Papuans: 5.77 × 10^9^/L vs. 6.86 × 10^9^/L, difference = -1.09 [(95% CI -0.42 to -1.79 × 10^9^/L), P = 0.0018]. 14 (9.2%) and 9 (5.9%) patients had leukopaenia (<4.0 × 10^9^/L) and leukocytosis (>10.0 × 10^9^/L), respectively. By Day 28, the mean WCC increased significantly (P = 0.0003) from 6.37 to 7.47 × 10^9^/L (73 paired values) and was similar between the two groups. Ethnicity was the only WCC explanatory factor and only on Day 0.

The mean Day 0 platelet count (n = 151) was 113.0 (range 8.0–313.0) × 10^9^/L and rose significantly to 186.308 × 10^9^/L by Day 28 (P < 0.0001). There was a corresponding fall in patient proportions with thrombocytopaenia (<150 × 10^9^/L): 119/151 (78.81%) vs. 16/73 (21.92%, P < 0.00001). Papuan and non Papuan mean platelet counts were similar at all time points. Only malaria species on Day 0 was a significant platelet count explanatory factor. The mean D0 platelet counts were significantly lower (P = 0.025) in vivax (102.022 × 10^9^/L) vs. falciparum (122.125 × 10^9^/L) patients.

**Conclusion:**

Changes in leukocytes and platelets were consistent with other malaria studies. The Papuan non Papuan difference in the mean Day 0 WCC was small but might be related to the difference in malaria exposure.

## Background

The malaria induced changes in the total white cell (WC) and platelet (PLT) counts have been documented in several clinical series of vivax and falciparum malaria in malaria immune and naïve patients of all ages [1-9], describing mostly the WC and PLT counts at presentation.

The majority of malaria patients at presentaiton have total WCCs in the normal range. Leukopaenia may affect up to 15% (WCC < 4 × 10^9^/L) of malaria infected adults [3,4,8,10] and was present in ~10% of 1,369 hospitalized Kenyan children with moderately severe or severe falciparum malaria (WCC < 6.1 × 10^9^/L) [6]. Rates of leukocytosis (>10 × 10^9^/L) in adults range between 1 and ~7% [1,3,4,9,11,12] and was 20% in the hospitalized Kenyan children (>16.5 × 10^9^/L) [6]. Reported, malaria induced changes in the differential white cell counts include neutropaenia, neutrophilia, immature neutrophils (left shift), neutrophil toxic granulation, lymphopaenia, lymphocytosis, atypical lymphocytes, monocytosis, eosinopaenia, post treatment eosinophilia, and leukaemoid reactions [3-5,10,11,13-19]. The underlying mechanisms include a shift in neutrophils from the circulatory to the marginal pool to sites of inflammation, splenic localisation, serum lymphotoxic factors, and intercurrent bacterial infections [13,15,20-23]. In experimentally induced falciparum malaria, Aotus monkeys had an increase in the absolute neutrophil counts seven days post infection that was followed by neutropaenia by Day 14; the reverse was found for the lymphocyte counts [24]. Ethnicity, sex and immune status may also be relevant factors affecting the leukocyte count. Leukocytosis was more common in *Plasmodium vivax *infected African American soldiers (27%) compared to Caucasian soldiers (9%) but the latter had higher rates (47 vs. 26%) of relative lymphocytosis (lymphocyte count > 35%) [14]. Studies in temperate climates have found that individuals of African origin tend to have lower total leukocyte, neutrophil and platelet counts compared to Caucasians and women have higher leukocyte and neutrophil counts than men [25,26]. Malaria naive Sri Lankans infected with *P*. *vivax *had higher mean lymphocyte counts and significantly raised gamma/delta T cells during fever paroxysms compared to malaria immune vivax patients, suggesting a greater inflammatory response by the non immune patients [27]. Similarly, TNFα levels were greater in non immune Sri Lankans with *P*. *vivax *[28].

Studies examining trends in the total WCC are few. One study of experimentally induced falciparum malaria in non immune Americans showed an initial fall in the mean WCC reaching a nadir on Day 3 and rising thereafter to baseline values by study end [2]. Studies in African children have shown modest falls in the mean total WCC over 7 to 14 days whilst another study found that acutely ill falciparum infected children had a significant fall in the mean neutrophil count on Day 3 [29-31].

Malaria induced thrombocytopaenia (platelet count < 150 × 10^9^/L) is very common, affecting between 40–85% of patients [4,7-9,32-35]. Moderate (50–100 × 10^9^/L) and severe (<50 × 10^9^/L) thrombocytopaenia occur in 30–50 and 6–16%, respectively [4,5,7,30,35]. Thrombocytopaenia improves with disease resolution and the platelet count is generally normal within seven days [34,35] but ranged from 2–28 days in one series [35]. Considerable overlap exists between the platelet counts of the different malaria species and between uncomplicated and severe falciparum malaria. Two studies suggest that a lower platelet count may be an indicator of higher biomass falciparum and vivax infections [36,37] but Eriksson *et al *did not find this correlation [4]. Factors associated with malaria induced thrombocytopaenia include splenomegaly, splenic sequestration and platelet removal by macrophages [38-40]. The role of antiplatelet antibodies is unclear [34,41,42].

In north east Papua, indigenous Papuans have life long exposure to malaria whereas Indonesians who immigrate to Papua usually contract malaria for the first time in Papua [43]. Whether this difference in malaria exposure affects the white cell and platelet counts in diseased patients is unknown. This paper presents data from a clinical trial on the evolution of the total white cell and platelet counts and explores possible Papuan non Papuan differences.

## Materials and methods

Malariometric data from this area of northeast Papua and study conduct are detailed elsewhere [44,45]. Briefly, indigenous adult Papuans (n = 57) and non Papuan (n = 105) adults from other parts of Indonesia were recruited into a hospital based, 28 days, clinical trial comparing chloroquine vs. chloroquine plus doxycycline vs. doxycycline for the treatment of parasitologically proven (Giemsa stained thick and thin blood films) acute uncomplicated falciparum and acute vivax malaria. There was no minimum parasitaemia for either species but a maximum of 150,000/μL asexual falciparum forms/μL for study entry. Recruited patients underwent a history, a detailed physical examination (including an abdominal examination to detect hepatosplenomegaly) and supervised treatment. Giemsa stained blood films were taken and read on Days 0–7, 14, 21, 28 and a blood count (Hb, total WCC, platelet count) was performed on Days 0, 3, 7, and 28; a manual differential white cell count was not done. Double entered, validated data were analysed using Epi Info 6.04b (Centers for Disease Control and Prevention, Atlanta, GA, USA) and Stata v 8 (Stata Corporation, USA). Normally distributed data were compared using the student's 't' test or ANOVA or the Mann-Whitney U or Kruskall-Wallis for skewed data. Spearman's rank correlation was used to determine the relationship between skewed continuous data. Proportions were compared using uncorrected chi squared values. Multivariate analyses were performed to assess the independence of ethnicity, splenomegaly, gender, malaria species and drug regimen on the total white cell and platelet counts. A P value of ≤ 0.05 was considered significant; no adjustment was made for multiple comparisons. Written informed consent was obtained from all patients. The study was conducted according to the Indonesian Ministry of Health, the Indonesian Navy, and the United States Navy and Army regulations governing the protection of human subjects.

## Results

A total of 162 malaria infected patients were recruited into the study: *P*.*vivax *(n = 63), *P*. *falciparum *(n = 89), and mixed infections (n = 10). Based on the dominant species, the 10 mixed infections were reclassified as vivax (n = 1) or falciparum (n = 9) malaria. Males (aged 15 to 44), numbered 146 (~90%), and women (aged 15 to 33) 16 (~10%). The Papuans numbered 57; the 105 non Papuans came from Java (n = 54), Sulawesi (n = 28), Ambon (n = 9), Bali (n = 7), Sumatera (n = 5), and other islands (n = 2). Median Papuan and non Papuan [interquartile (IQ) range] residential times in Papua were 21.5 (18 to 24) and three (1 to 5) years, respectively (P < 0.0001). 152/162 (93.8%) had blood results available for Day 0 and 74 patients completed the study to Day 28. Splenomegly was more common in the Papuans, 26/57 (45.6%), compared to the non Papuans, 27/105 (25.7%), for a relative risk of 1.8 [95% confidence interval (CI) 1.15 to 2.7].

### Day 0 total white cell counts

The Day 0 total WCCs ranged from 2.1–13.4 × 10^9^/L with a median and mean of 6.3 and 6.492 × 10^9^/L, respectively. Leukopaenia (WCC < 4 × 10^9^/L) was present in 14 patients (9.2%); their WCCs ranged from 2.1–3.9 × 10^9^/L. Nine (5.9%) patients had a leukocytosis (WCC > 10 × 10^9^/L). Thus, the majority of patients, ~85%, had a normal total WCC at presentation. In the multivariate analysis, only ethnicity was a significant explanatory variable for the total WCC on Day 0. The mean Day 0 total WCC was significantly lower in the Papuans (5.7 × 10^9^/L) compared to the non Papuans (6.8 × 10^9^/L): difference = -1.09 [(95% CI -0.42 to -1.79 × 10^9^/L), P = 0.0018]. There was a positive correlation between the Day 0 WCC and the *P*. *vivax *parasitaemia (Sp rho = 0.43, P = 0.0003) but no correlation between the Day 0 WCCs and: (i) the Day 0 falciparum counts [Spearman's (Sp) rho = 0.139, P = 0.19], (ii) the Day 0 temperature (Sp rho = -0.0066, P = 0.94), and (iii) the Day 0 platelet count (Sp rho = 0.125, P = 0.12).

### Evolution of the total white cell counts

**Figure 1:**
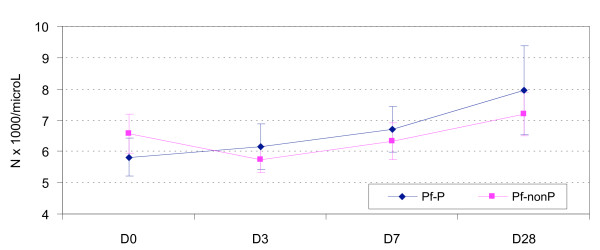
**The mean (95% CI) total white cell counts in falciparum infected Papuan and non Papuan patients**.

**Figure 2:**
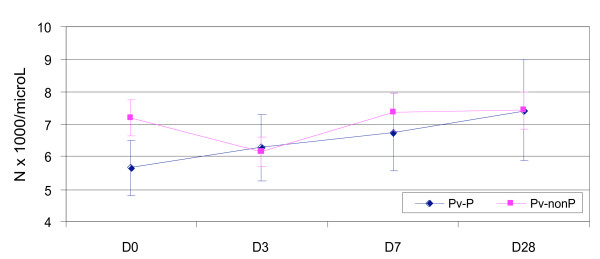
**The mean (95% CI) total white cell counts in vivax infected Papuan (5.66 × 10^9^/L) and non Papuan (7.20 × 10^9^/L) patients**. The mean difference at baseline is -1.54 (-0.47 to -2.62) × 10^9^/L, P = 0.0055].

The changes in the mean white cell counts during follow up were similar for both malaria species and ethnic groups (Figures 1 and 2). Compared to baseline, the fall in the mean total WCC on Day 3 was significant in the non Papuans: (i) Pf: -0.898 (5.735 – 6.633) × 10^9^/L (P = 0.0015), (ii) Pv: -1.069 (6.132 – 7.201) × 10^9^/L (P = 0.0012). The mean Day 3 Day 0 difference between the non Papuans and Papuans was also significantly different [Pf: -0.898 vs. 0.248 × 10^9^/L (P = 0.008), Pv: -1.069 vs. 0.626 × 10^9^/L (P = 0.0054)].

By Day 28, the number of evaluable patients had fallen to 74. The mean total WCC was: (i) significantly higher compared to baseline (n = 73 pairs) 7.47 vs. 6.37 × 10^9^/L: difference = 1.1 (0.52 – 1.69) × 10^9^/L, P = 0.0003] and (ii) similar between the Papuans [7.79 × 10^9^/L (n = 27)] and the non Papuans [7.3 × 10^9^/μL (n = 47)] but there was a trend towards a higher mean change in the Papuans compared to the non Papuans: 1.8 vs. 0.7 × 10^9^/L (P = 0.07). There was no Day 28 leukopaenia but five (6.7%) patients had leukocytosis. Their Day 28 Day 0 matching values (x 10^9^/L) were: 10.4–3.4, 10.8–6, 12–7.2, 12.2–9.2, 16.4–7. They were all aparasitaemic, afebrile, and discharged clinically well from the hospital.

### Day 0 platelet counts

The Day 0 platelet counts (PLT0) for all patients combined varied widely, ranging from 8–313 × 10^9^/L (IQ range 76 to 141 × 10^9^/L); the median and mean values were 102 and 113 × 10^9^/μL, respectively. The majority of patients, 119/151 (78.8%), had thrombocytopaenia (<150 × 10^9^/L); 43.7% (66/151) had moderate thrombocytopaenia (50–100 × 10^9^/L). Five *P*. *falciparum *and eight had *P*. *vivax *(8.6%) infeted patients were severely thrombocytopaenic (<50 × 10^9^/μL) but none had clinical signs of bleeding. Of the 88 patients who had urine dipstick tests, nine (10.2%) had microscopic haematuria (vivax = 4, falciparum = 5); the platelet counts in these nine ranged from 50–313 (median 101) × 10^9^/L.

The multivariate analysis showed that only the malaria species was a significant independent variable to explain the Day 0 platelet count. The mean PLT0 was significantly lower (P = 0.025) in the vivax patients: 102.022 vs. 122.125 × 10^9^/L. The PLT0 was negatively correlated with the Day 0 falciparum parasitaemia (Sp rho = -0.29, P = 0.005) but not with the Day 0 vivax parasitaemia (Sp rho = -0.0984, P = 0.4). There was also a significant negative correlation with the Day 0 temperature (Sp rho = -0.21, P = 0.009) and the PLT0 in the vivax patients (Sp rho = -0.26, P = 0.04) and a negative trend in falciparum patients (Sp rho = -0.18, P = 0.097).

### Evolution of the platelet counts

**Figure 3:**
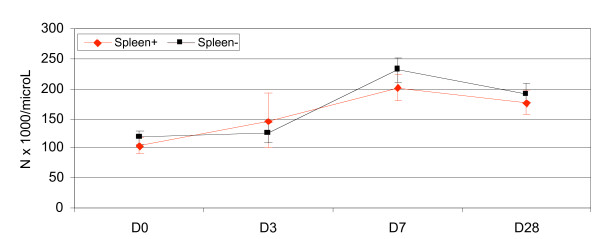
**Mean (95% CI) platelet counts of malaria infected Papuans and non Papuans as a function of a palpable spleen**.

**Figure 4:**
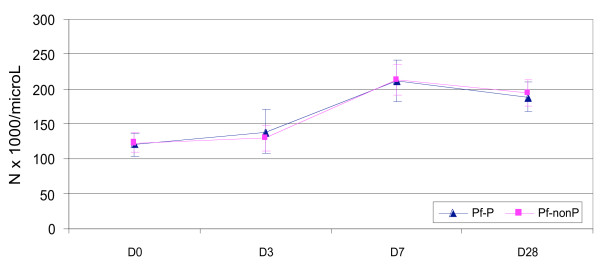
**The mean (95% CI) platelet counts in falciparum infected Papuan and non Papuan patients**.

**Figure 5:**
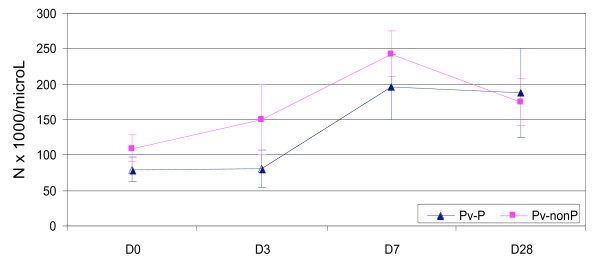
**The mean (95% CI) platelet counts in vivax infected Papuan and non Papuan patients**.

The proportion of thrombocytopaenic patients declined over time: 78.8% (119/151) on D0, 74.26% (101/136) on D3, 23.62% (30/127) on D7 and 21.92% (16/73) on Day 28 (P < 0.0001, chi^2 ^for trend). By Day 28, the mean platelet count was significantly higher compared to baseline: 188.308 vs. 108.056 × 10^9^/L (P < 0.0001), and ranged from 47.0 to 318.2 (IQ range 153 to 226) × 10^9^/L (Figures 3, 4 and 5). The mean D28-D0 changes in platelet counts were not significantly different between the Papuans and non Papuans: 83.280 vs. 75.576 × 10^9^/L (P = 0.6). The mean Day 7 platelet count was significantly higher than that of Day 28 only in the vivax infected non Papuans (n = 21): 175.819 vs. 251.238 × 10^9^/L (P = 0.0036). The multivariate analyses were unremarkable, except for drug arm as a PLT7 explanatory factor. This was accounted for by the combination vs. the doxycycline arm: 235.463 vs 191.741 × 10^9^/L (P = 0.029).

## Discussion

These analyses have shown that at presentation the majority of malaria infected patients had total white cell counts within the normal range and most were thrombocytopaenic. By Day 28, both mean counts had increased but thrombocytopaenia remained in just over 20% of patients. These data complement and are broadly consistent with those of other clinical series.

Data from this study were from two dissimilar groups with respect to ethnicity and the degree of malaria acquired immunity. The Papuans had life long malaria exposure whilst the non Papuans had limited or no malaria exposure. The Papuans had lower mean Day 0 white counts than the non Papuans and there was a notable difference in the WCC changes over time. They rose steadily in the Papuans but fell significantly on Day 3 in the non Papuans, consistent with a study of experimentally induced falciparum malaria in malaria naïve American volunteers [2]. By Day 28, the mean WCCs in the non Papuans had caught up and both groups had similar mean WCCs. This difference in the WCC progressions between the two ethnic groups is difficult to explain purely on the basis of a redistribution of white cells and/or splenic sequestration, given that the malaria naïve non Papuans had significantly less splenomegaly. These data might indicate that a lesser degree of malaria exposure, thus acquired immunity, in the non Papuans resulted in a more robust inflammatory response, reflected crudely by a higher, baseline total WCC [27]. The initial fall in the total WCC in the non Papuans might reflect a difference in response to dying parasitized red cells consequent to treatment.

Thrombocytopaenia was common at presentation in both groups of patients. There were no differences between the Papuans and non Papuans but the mean platelet count was lower in vivax compared to falciparum infected patients. There were negative correlations between the Day 0 platelet count and the falciparum parasite count and the Day 0 temperature in the vivax patients. These findings are consistent with some but not other reports and are of limited clinical value [2,4,8,31,34]. Over time, the mean platelet count increased, consistent with the findings of others but two reports document an initial fall in the platelet count followed by recovery [2,4]. The mean platelet count appeared higher on Day 7 compared to Day 28 but there are no intervening platelet counts, so the day of the mean peak platelet count cannot be determined. Some 20% of patients were still thrombocytopaenic by study end, suggesting a number of patients need longer than four weeks for full platelet recovery. The multivariate analysis found drug arm to be significant factor for the Day 7 platelet count with the combination arm having a higher mean platelet count than the doxycycline arm. This is of little clinical significance.

Our study had limitations. The study sample was small and powered for a clinical trial. The data presented were secondary analyses to look for possible Papuan non Papuan differences. Many patients failed treatment before Day 28, given rescue treatment and withdrawn from the study; this may have introduced a statistical bias. Most patients were male, thus limiting the applicability of the findings. There were many statistical comparisons and some significant results may have occurred by chance. The differential white cell count was not measured and could have provided additional interesting data.

## Conclusion

To conclude, these analyses have compared and contrasted the changes in the total white cell and platelet counts in Papuans and non Papuans with different degrees of malaria exposure that may partly explain the difference in the mean total white cell count at presentation between the two groups.

## Authors' contributions

WRJT, the principal investigator, developed the protocol, supervised study execution, analysed the data and wrote the first draft of the paper. CO conceived the study, developed the protocol and criticially reveiwed the manuscript. ET developed the protocol, was involved in study execution and critically reviewed the manuscript. SLH developed the protocol and critically reviewed the manuscript. TLR devleoped the protocol and criticially reviewed the manuscript. HW played a key role in study execution and reviewed the manuscript. HB was involved in study execution and reviewed the manuscript. Taufik was involved in study execution and reviewed the mauscript. SB was inolved in study execution and reviewed the manuscript. DF was involved in data interpretaiton and crticially reviewed the manuscript.
